# Free Sugars Intake among Chinese Adolescents and Its Association with Dental Caries: A Cross-Sectional Study

**DOI:** 10.3390/nu13030765

**Published:** 2021-02-26

**Authors:** Qiping Yang, Yue Xi, Hanmei Liu, Jing Luo, Yufeng Ouyang, Minghui Sun, Cuiting Yong, Caihong Xiang, Qian Lin

**Affiliations:** Department of Nutrition Science and Food Hygiene, Xiangya School of Public Health, Central South University, 110 Xiangya Rd, Changsha 410078, China; yangqiping12@csu.edu.cn (Q.Y.); xiyue0404@csu.edu.cn (Y.X.); hanmeiliu@csu.edu.cn (H.L.); luojing2546@csu.edu.cn (J.L.); oyyf0102@csu.edu.cn (Y.O.); sun.1234@csu.edu.cn (M.S.); yongcuiting@csu.edu.cn (C.Y.); xch0622@csu.edu.cn (C.X.)

**Keywords:** adolescent, dietary sugars, sugar-sweetened beverages, dental caries, oral health

## Abstract

This study aims to estimate the free sugars intake, identify the primary food sources of free sugars, and explore the relationship between free sugars intake and dental caries among Chinese adolescents. This cross-sectional study included 1517 middle-school students aged 12–14 years in Changsha city, China. Adolescents completed a 12-item Food Frequency Questionnaire (FFQ) and oral health assessment. The students’ dental caries experience was available as DMFT score (number of decayed, missing, and filled permanent teeth). Statistical analyses included the Mann–Whitney test, Kruskal–Wallis test, Chi-square test, and binary logistic regression model. The average intake of free sugars was 53.1 g/d in adolescents, and 43.2% of the students consumed more than 50 g of free sugars daily. The primary contributor to free sugars was sugar-sweetened beverages (SSBs). Age, boarders, and high family income were risk factors for excessive free sugars intake (*p* < 0.05), and increased free sugars intake was a risk factor for dental caries (odds ratio, OR = 1.446, 95% confidence interval: 1.138–1.839). Both the free sugars intake and dental caries prevalence in Chinese adolescents were high. Targeted interventions are urgently needed to address the excessive consumption of free sugars and improve Chinese adolescents’ oral health.

## 1. Introduction

Dental caries is one of the most prevalent oral conditions worldwide, which affects about 35.3% of the global population at all ages [1]. Tooth decays begin for many people from early stages without symptoms and continue at advanced settings causing pain, anxiety, functional limitation (including low school attendance and performance in children), and social handicap through tooth loss [2]. Not only does caries harm individuals’ health, but it results in a health-related economic burden. It has been estimated that 298 billion USD was spent on direct costs associated with dental caries, accounting for about 67.4% of the total financial outlay globally in 2010 [3]. In 2017, dental caries affected 621 million children worldwide [3], with a 20.6% rise during a decade [4]; as for China, the prevalence of permanent teeth caries among 12-year-olds increased by 7.8% from 2007 to 2017 [5]. This increase, at home and abroad, alarmed us to pay more attention to dental caries prevention. Dental caries is mostly preventable through cost-effective and straightforward population-wide and individual interventions [2].

Dental caries is significantly moderated by age, sex, social determinants (income and education), health-related behaviors (smoking, diet, alcohol consumption, and healthcare attendance), and water fluoridation [6]. Sugars are the essential factor for dental caries [7]; a decayed tooth develops when bacteria in the mouth metabolize sugars to produce acid that demineralizes the tooth’s hard tissues. As defined by the World Health Organization (WHO) in 2015, free sugars include monosaccharides and disaccharides added to foods and beverages by the manufacturer, cook, consumer, and sugars naturally present in honey syrups, fruit juices, and fruit juice concentrates. Free sugars contribute to diets’ overall energy density and promote a positive energy balance [8,9]. Due to the association between free sugars intake and risk of dental caries [10], the increasing free sugars intake, especially in the form of sugar-sweetened beverages (SSBs) and sweetened foods, is of concern among children [11], adolescents [12,13], and adults [14]. Since the sweet preference is higher in adolescence than in adulthood [15,16], this stage is particularly vulnerable to the health consequences of high sugars intake. In several foreign studies [17,18,19,20], a large proportion of adolescents exceeded the recommendations on free sugars consumption. Such a high sugars intake may be of relevance, as adolescence is suggested to be a “critical period” for developing various diseases in later life [21,22], and dietary patterns have been shown to track into adulthood. However, the relevant studies conducted on Chinese adolescents are limited, and they mostly focused on the intake frequency of sugar-sweetened beverages [23,24,25] but ignored the amount of total free sugars consumption.

To date, no studies have explored the association of free sugar intake with dental caries among Chinese adolescents, and the amount and source of their free sugars’ consumption is unclear. Therefore, this study aims to assess the daily intake of free sugars as well as identify the food sources of free sugars and the association between free sugars’ consumption and dental caries in Chinese adolescents aged 12–14 years. We hypothesized that the risk of dental caries is positively associated with the consumption of free sugars. This study may be helpful for developing health strategies concerning free sugar intake and oral health for Chinese adolescents.

## 2. Materials and Methods

### 2.1. Ethical Approval

The study was approved by the Ethics Review Committee of the Xiangya School of Public Health, Central South University (XYGW-2019-025). Before the investigation, written informed consent from parents or caregivers was obtained, and all information was kept confidential.

### 2.2. Study Design and Participants

This cross-sectional study was conducted from March to July 2019 in Changsha, the capital city of Hunan Province in south-central China. Two-stage stratified random cluster sampling was used to select ten middle schools from five districts (Yuhua District, Tianxin District, Furong District, Kaifu District, and Yuelu District) in Changsha city. In each section, two middle schools were selected randomly. Recruitment was targeted at 7th and 8th-grade students. The inclusion criteria for selection are as follows: (1) schools that enrolled more than 500 students and agreed to participate in the study, (2) parents’ or caregivers’ information consented and agreed to participate. Exclusion criteria were those students who could not read or write and thus complete the questionnaire.

Before the investigation, we submitted the protocol to the local Education Bureau for permission to conduct this investigation. Under the education bureau’s assistance, we contacted leaders of the selected schools and delivered consent forms to students. The eligible students were enrolled if their parents or caregivers signed informed consent. A total of 1628 students participated in this study; of these, 1517 students finished the questionnaire and were included in the final analyses (participate rate: 93.2%).

### 2.3. Measures

The survey included an online questionnaire, anthropometric measurements, and dental caries assessment. Teachers in the selected schools organized all the processes.

#### 2.3.1. Questionnaire Survey

Uniformly trained research assistants guided the students to complete the online questionnaire in the school computer room. The online questionnaire was delivered via The Questionnaire Star, which is a tool used to develop electronic questionnaires. Each questionnaire had a unique linkage. Along with demographics information, students’ food consumption and oral health-related behaviors were collected.

- Students’ demographic information: including students’ sex, age, ethnicity, sibling status (the only child or not), family monthly income, and parents’ education attainment.
- Food consumption: A 12-item Food Frequency Questionnaire (FFQ) was used to assess food consumption during the last week. This FFQ consisted of cereals and wheat, vegetables (leafy vegetables, melon vegetables, and root or stem vegetables), fruits, soybeans and its products, dairy and its products, meat, poultry, fish and shrimp, eggs, and SSBs and sweetened foods. The 6-category response options of intake frequencies ranged from “once a month or less” to “twice or more a day”.
- SSBs and sweetened foods consumption: The average intake of SSBs and sweetened foods were collected via the FFQ mentioned above. The food groups were set according to database of free sugars content from Chinese Center for Disease Control and Prevention (China CDC) (shown in Table A1). Carbonated drinks, vegetable protein drinks, juice or juice drink, tea drinks, sports drinks, and bubble tea were included in SSBs. Sweetened foods included cakes, desserts, confectionery (chocolate, Snickers and Maltesers, etc.), and preserved fruits (dried fruits and candied fruits). Honey and flavored milk/yogurt were also included as sources of free sugars. The response options of the average intake of SSBs and sugary foods included “100 mL/time, 200 mL/time, 300 mL/time, 400 mL/time and 500 mL/time” and “25 g/time, 50 g/time, 75 g/time, 100 g/time, 150 g/time and 200 g/time”, respectively.
- Oral health-related behaviors: Oral health-related behaviors were collected by five self-designed questions, including: (1) Do you have the dental health education lectures in your school? (2) How often do you brush your teeth every day? (with the response option of “1 time/day or less” and “2 times/day or more”) (3) Will you brush your teeth after intake of SSBs and/or sweetened foods? (4) Do you often use dental floss or mouthwash? (5) Do you have a regular dental visiting?

#### 2.3.2. Anthropometric Measurements

Each participant’s height and weight were measured by uniformly trained research assistants using the standard height meter and TANITA human body composition analyzer BC-W02C (Guangdong Food and Drug Administration (prospective), No 2210704, 2014). Body mass index (BMI) was calculated via dividing body weight (kg) by height squared (m^2^). According to age- and sex-specific BMI cut-off values for Chinese children and adolescents (6–18 years old) [26], participants were classified as in either the normal or overweight/obese group (shown in Table A2).

#### 2.3.3. Dental Caries Assessment

Dental caries conditions were evaluated and recorded by two professional dentists. Students lined up in a classroom. The dentists assessed their oral hygiene status one by one using a disposable dental mirror with a light-emitting diode (LED) light and ball-end Community Periodontal Index (CPI) probe. Food debris was gently removed to avoid the under-recording of dental caries. The diagnostic criteria for dental caries followed the recommendation from the WHO [27].

- Dental caries experience was measured via the decayed–missing–filled teeth (DMFT) index. DMFT is a cumulative measurement by summing the number of decayed (D), missing (M), and filled (F) teeth. A tooth that decayed but has not been filled would be recorded as decayed (D). When a tooth was extracted due to caries, it would be recorded as missing (M). A tooth recorded as filled (F) when it was permanently filled without caries.
- Dental caries prevalence, the proportion of the students with the DMFT ≥ 1, was set as a dichotomous dependent variable. After the dental examination, an individual report of oral hygiene was delivered to each student.

### 2.4. Free Sugars Intake Assessment

The primary sources of free sugars were derived from SSBs and sweetened foods included in the 12-item FFQ mentioned above. The response options of FFQ were converted to daily consumption frequencies as follows: 0 = “once a month or less”, 0.14 times/day = “once a week”, 0.36 times/day = “2–3 times a week”, 0.64 times/day = “4–5 times a week” and 2 times/day = “2 times/day or more”.

The following Formula (1) calculated the individual’s estimated daily intake of free sugars:

(1) $$Z=A\left(f_{1}\right)\times \frac{A\left(i_{1}\right)}{7}\times c_{1}+B(f_{2})\times \frac{B\left(i_{2}\right)}{7}\times c_{2}+\ldots +X(f_{n})\times \frac{X\left(i_{n}\right)}{7}\times c_{n}.$$

Daily consumption of free sugars (Z, g/d); food types (A, B,⋯, X); frequency (f, times/d); intake of each time (*i*, g or mL); and free sugars content were from the China CDC database (c, g/100 g or mL/100 mL. Shown in Table A1), and n is a natural number. For some certain foods absent from the database, the average sugar content of such types of foods was used as a substitution. According to the recommendation of the latest China’s dietary guidelines [28], students were divided into low-sugar consumption groups, medium-sugar consumption groups, and high-sugar consumption groups (25 g and 50 g as the cut-off values, respectively).

### 2.5. Statistical Analyses

EpiData3.0 software (The Epi Data Association, Odense, Denmark) was used for data entry, the IBM SPSS24.0 software (IBM Corp., Armonk, NY, USA) was used for data analyses, and the figure was presented by GraphPad Prism7.0 (GraphPad Software, Inc., San Diego, CA, USA). Descriptive information was presented as percentage or the mean with standard deviation. Chi-squared test and non-parametric tests were used to analyze general demographic data for different classification variables or continuous variables. Binary logistic regression analysis was used to analyze the association between free sugars intake and dental caries, adjusted by sex, age, BMI status, domicile place, and regular dental visiting. Significant levels were set at *p* < 0.05.

## 3. Results

### 3.1. Characteristics of Participants

A total of 1517 students in 7th and 8th grades from ten schools participated in this study (Table 1). Of these, 53.3% were boys. Ages ranged from 12 to 14 years old, and most students were 12 years old (47.3%). Only children accounted for 44.7% of the total adolescents. Most of the students (92.2%) lived in their own homes during the school years. Overall, there were 27.3% of the students who were overweight/obese. More than half of the students’ parents (65.2%) had acquired high school education or above. There were 39.8% of the students whose household incomes were lower than 5000 *RMB* monthly. The average intake of free sugars was 53.1 g/d in the present study. The higher the students’ grades, the more the free sugars intake (56.3 g/d vs. 50.2 g/d, *p* < 0.05). The consumption of free sugars in boarding students was significantly higher than that of the students who lived in their own homes during the school year (62.4 g/d vs. 52.4 g/d, *p* < 0.05).

The prevalence of dental caries was 56.9%, and the weighted mean DMFT was 1.45 (95% CI: 1.36–1.54). More girls than boys had dental caries (62.3% vs. 52.1%, *p* < 0.05), and the prevalence of dental caries among the only children was lower than that of those with siblings (53.7% vs. 59.5%, *p* < 0.05). Compared to the students with normal BMI status, those who were overweight/obese had a lower prevalence of dental caries (58.9% vs. 51.4%, *p* < 0.05), as well as a lower risk of dental caries experiences (1.55 vs. 1.20, *p* < 0.05). More adolescents from the rural area than the urban area had a higher prevalence of dental caries (55.1% vs. 60.4%, *p* < 0.05). The students who visited dentists regularly had both a higher prevalence of dental caries (63.5% vs. 55.1%, *p* < 0.05) and risk of dental caries experiences (1.67 vs. 1.39, *p* < 0.05).

### 3.2. Free Sugars Intake and Its Sources among Adolescents

**Figure 1:**
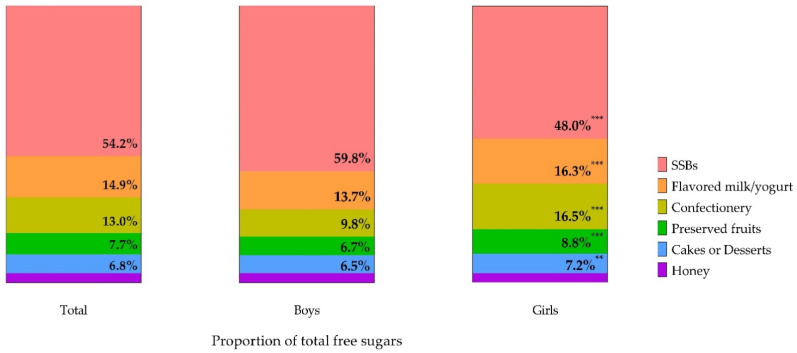
Sources of free sugars for adolescents by sex (the proportion of total free sugars, %). * *p* < 0.05, ** *p* < 0.01, *** *p* < 0.001; compared boys with girls by Mann–Whitney U test. Sugar-sweetened beverages (SSBs) include sodas, vegetable protein beverages, juice or juice drinks, tea drinks, sports drinks, and bubble tea.

According to *The Dietary Guidelines for Chinese Residents (2016)*, the appropriate daily intake of added sugars for adults should not exceed 50 g, and it would be preferable if intake was below 25 g. In our study, only 30.6% of adolescents consumed free sugars lower than 25 g/d, and 43.2% of adolescents had an excessive intake of free sugars higher than the recommended maximum level (Table 1). The primary source of free sugars in the adolescents was SSBs, which provided 54.2% for free sugars’ daily intake (Figure 1). Flavored milk/yogurt and confectionery followed, with the contribution of 14.9% and 13.0%, respectively, which were followed by preserved fruits, cakes or desserts, and honey. For sex, boys consumed more SSBs than girls did, while girls’ intake of confections, flavored milk/yogurt, preserved fruits, and cakes and desserts were all higher than boys’ (*p_-all_* < 0.05).

### 3.3. Association between Free Sugars Consumption and Dental Caries

In the present study, 56.9% of the students had dental caries. Adolescents with decayed teeth consumed more free sugars than those without dental caries (56.9 g/d vs. 48.2 g/d, *p* < 0.05). For specific food groups, the differences between the adolescents with and without dental caries were similar to the consumption of free sugars in different sex (Table 2) and weekly frequencies (Table A3).

The binary logistic regression model was also conducted to evaluate the association between dental caries as the dependent variable (DMFT = 0 as the reference) and free sugars intake (Table 3). Our results found that the students who consume free sugars more than 50 g/d were significantly more likely to have dental caries (crude OR = 1.446, 95% CI: 1.138–1.839, *p* < 0.05). These associations remained after the adjustment. For different sources of free sugars, a higher intake of SSBs (adjusted OR = 1.005, 95% CI: 1.002–1.007, *p* < 0.05), confections (adjusted OR = 1.005, 95% CI: 1.000–1.010, *p* < 0.05), flavored milk/yogurt (adjusted OR = 1.007, 95% CI: 1.000–1.014, *p* < 0.05) and honey (adjusted OR = 1.015, 95% CI: 1.001–1.029, *p* < 0.05) were risk factors of suffering dental caries. Meanwhile, there were no associations between preserved fruits, cakes and desserts, and dental caries.

## 4. Discussion

Our study indicates that 12–14-year-old Chinese adolescents have a high consumption of free sugars, which are associated with the prevalence of dental caries. Adolescents who are female, more senior, and living in the dormitory during school years tend to consume more SSBs and sugary foods and thus might have a higher risk of dental caries. Given that the information on the consumption of free sugars in China is insufficient, this study would guide sugar intake reduction and dental caries prevention in adolescents.

In our results, the free sugars intake of adolescents was 53.1 g/d, which is lower than that in U.S. adolescents with 94.0 g/d [19], Latin American countries from 58.2 to 106.9 g/d [20], and in European countries with 110.1 g/d [29]. This may be due to urbanization levels, local dietary habits, measuring and analytical methods of free sugars in different countries [30]. Studies on the consumption of free sugars in adolescents from China are limited. Compared with other age groups, the average intake of free sugars in adolescents is higher than that of adults who consumed sugary foods in China (18.8 g/d) [31], and it is also higher than that of residents in Tianjin City with an intake of 24.1 g/d [32]. Several studies suggested that the consumption of free sugars topped in adolescents [30,33], which was reflected by our findings that older students consumed more free sugars than 12-year-olds did. This could be explained by their immaturity in choosing their foods [30,34], and largely by the influence of many factors such as living environments and the availability of sugary drinks [35,36,37]. It is also supported by our results that the boarders consumed more free sugars daily than day students. Overall, public health programs to lower sugar intake need to focus on this vulnerable population urgently.

We also found the main contributor to free sugars in adolescents was SSBs (54.2%). Similar findings were observed in other countries [18,38,39,40]. However, in our study, the categories that followed SSBs were flavored milk/yogurt (14.9%) and confectionery (13.0%), which was slightly different from other studies. Among Australian adolescents, sugars and sweet spreads, cakes, biscuits, pastries, and batter-based products were the secondary sources of free sugars [39]. In Spain, except for SSBs, the major source of free sugars intake in children and adolescents were sugar and bakery and pastry items [41]. For Greek 10–12-year-old adolescents, sweets, sugared fruit juices, and chocolate milk were major contributors apart from SSBs [42]. This could be explained by regional disparities, such as related policy [43], economic level [34], food consumption habits [34], and variations of food composition [44]. In line with the previous studies, we also found that boys tend to drink more sugary drinks [30,45,46], while girls prefer sugary foods; this could be because of adolescents’ sweetness and food preference [15], physical activity [47], as well as pubertal desires for a slim body [46]. A reduction of SSBs consumption is urgently needed in adolescents. It is essential to improve schools’ food environment by regulating the sale of beverages and foods that are high in free sugars [48].

Given China’s thriving production and output of SSBs [49,50], the impact of excessive free sugars intake on adolescents’ health should be of concern. Dental caries is one of the most known consequences of an excessive intake of free sugars. Our study presented that the prevalence of dental caries in adolescents was at a high level of 56.9%, which was not only higher than the 34.5% from the *4th National Oral Health Epidemiological Survey* [5] but also higher than that of students in Jiangxi province and Zhejiang province (25.8% and 44.0%, respectively) [51,52]. According to the assessment criteria of DMFT from WHO [27], average caries (DMFT) in 12-year-olds ranged from 1.2 to 2.6, representing a generally low level. Our study revealed that the mean caries (DMFT) of the adolescents was 1.45 (95% CI 1.36–1.54), which was higher than the overall caries of 12–15-year-old teenagers in Jiangxi province (0.48) [51], Chongqing Province (0.99) [53], and the national level (1.04) [5]. However, it was lower than rural left-behind children aged 12 years old in Henan province with a DMFT of 1.75 [54]. Both the dental caries score (DMFT) and dental caries prevalence of female students were higher than those of male students, which was in accordance with previous findings [51,52].

With Chinese characteristics, we found only children had a lower DMFT as well as the prevalence of dental caries. Compared with children with siblings, the only children are more likely to be well brought up and supervised [51]. However, we did not observe the association between low family income, low parental education attainments, and dental caries. It might be contrary to the major viewpoints that people with low socioeconomic status might have poor oral status [55] but could be explained by the universal coverage of medical insurance in China [56]. Furthermore, health education lectures held in school might decrease the impact of parental socioeconomic status on oral health. Rather than accelerating economic development, it is more important to disseminate health messages. Health professionals and teachers have a crucial role in such nutrition health education on sugars and health in school [7].

Apart from oral health practices, fluoride is another preventive factor for dental caries. Although the use of mouth rinses was considered, the use of fluoridated toothpaste or mouth rinses was not evaluated in our study. Some studies have found that even considering the use of fluoride, the effect of sugar intake on dental caries still exists [57,58]; these findings highlighted the need for low sugars intake throughout life, whether fluoride intake is optimum [59]. Above all, verifying the association between free sugars intakes and dental caries would be practical for policy makers in China to adopt multiple measures to reduce the free sugars intake in adolescents.

We did not observe the associations between BMI and free sugars intake among adolescents, which was different from other studies [45,60,61]. Individuals with overweight/obesity are more likely to underreport their consumption and provide socially desirable answers, particularly in adolescents [62]. Another probability for this result was that we could not adjust energy intake, while it is well known that excess energy intake plays a crucial role in weight gain [63]. This study found a negative association between BMI status and dental caries, which is in accordance with some research [64,65,66] but inconsistent with the majority [67,68,69]. Individuals, especially overweight or obese adolescents, tend to restrict their intake from high-fat and high-sugar foods [70], while lean, active individuals tend to select high-energy and high-sugar diets [71]; thus, low caries is associated with high BMI. Another explanation may be an increased consumption of high-fat diets, which is associated with weight gain positively, rather than caries [72].

The main strengths of our study are its sufficient sample size and strict quality control. The oral status was assessed and recorded by the professional dentists, and the weight status was measured by uniformly trained assistants, which improved the reliability of the anthropometric data. Moreover, the investigation tool was reliable, and participants had to complete it before submitting it to ensure the integrity of the questionnaire data.

However, there were several limitations. Firstly, the causal relationship between free sugars intake and dental caries could not be identified due to the cross-sectional study. For another, the uses of fluoridated toothpaste or mouth rinses among the participants could not be assessed in this study. Moreover, we used a semi-quantitative FFQ, which could not accurately reflect the intake of free sugars since the total energy intake could not be estimated and adjusted. In addition, some free sugars were not included, such as sucrose in homemade dishes, or the free sugars in some foods is unknown. Additionally, the study sample was from Changsha, the Hunan Province’s capital city in central-south China. Therefore, the findings cannot be extrapolated to adolescents in rural or other parts of China, where the eating habits and dietary culture are different.

## 5. Conclusions

In addition to the excessive consumption of free sugars, the prevalence of dental caries in Chinese adolescents was relatively high. The primary source of free sugars for teenagers was sugar-sweetened beverages, and free sugars intake is associated with a high risk of dental caries. Promoting a healthy eating environment and restricting SSBs consumption would be important for addressing excessive intake of free sugars for adolescents. Future research should focus more on developing the free sugars database to estimate sugar intake conveniently. Simultaneously, a prospective study of a large population should be conducted to clarify the causal association between free sugars and dental caries or other diseases.

## Figures and Tables

**Table 1:** Characteristics of the adolescents by outcome variables (*n* = 1517).

Variables	N (%)	Daily Intake of Free Sugars	Dental Caries (DMFT ≥ 1)	DMFT
		(g/d, Mean ± *S.D.*)	*n* (%)	Weighted Mean (95% CI)
Total samples	1517 (100)	53.1 ± 44.8	863 (56.9)	1.45 (1.36, 1.54)
Sex				
Boy	808 (53.3)	54.5 ± 44.1	421 (52.1) ***	1.25 (1.14, 1.36) ***
Girl	709 (46.7)	51.6 ± 42.8	442 (62.3)	1.68 (1.55, 1.81)
Age (y)				
12	396 (26.1)	48.3 ± 43.1 *	228 (57.6)	1.54 (1.35, 1.72)
13	717 (47.3)	54.4 ± 45.4	408 (56.9)	1.44 (1.31, 1.56)
14	404 (26.6)	55.8 ± 45.0	227 (56.2)	1.39 (1.23, 1.55)
Grade				
7th grade	784 (51.7)	50.2 ± 44.2 **	449 (57.3)	1.49 (1.36, 1.61)
8th grade	733 (48.3)	56.3 ± 45.1	414 (56.5)	1.41 (1.29, 1.53)
Only child				
Yes	678 (44.7)	52.9 ± 44.9	364 (53.7) *	1.34 (1.21, 1.46) *
No	839 (55.3)	53.4 ± 44.7	499 (59.5)	1.54 (1.42, 1.66)
BMI status				
Normal	1103 (72.7)	54.6 ± 45.8	650 (58.9) **	1.55 (1.44, 1.65) ***
Overweight/obese	414 (27.3)	49.2 ± 41.7	213 (51.4)	1.20 (1.04, 1.35)
Ethnicity				
Han	1448 (95.5)	53.3 ± 44.8	828 (57.2)	1.46 (1.37, 1.55)
Minorities	69 (4.5)	50.3 ± 44.7	35 (50.7)	1.29 (0.87, 1.71)
Domiciled place				
City	1014 (66.8)	53.6 ± 45.3	559 (55.1) *	1.40 (1.29, 1.50)
Countryside	503 (33.2)	52.2 ± 43.7	304 (60.4)	1.56 (1.41, 1.72)
Live in or out				
Dormitory	118 (7.8)	62.4 ± 50.1 *	77 (65.3)	1.53 (1.25, 1.82)
Own home	1399 (92.2)	52.4 ± 44.2	786 (56.2)	1.44 (1.35, 1.54)
Family monthly income				
Low	408 (39.8)	51.2 ± 44.6	236 (57.8)	1.45 (1.28, 1.62)
Middle	370 (36.0)	53.9 ± 43.0	219 (59.2)	1.51 (1.34, 1.69)
High	248 (24.2)	54.9 ± 45.9	131 (52.8)	1.40 (1.18, 1.63)
Parents’ education attainment				
Primary school or blow	66 (5.1)	52.5 ± 46.3	39 (59.1)	1.30 (0.92, 1.68)
Middle school	380 (29.7)	55.9 ± 47.3	211 (55.5)	1.44 (1.27, 1.61)
High school	459 (35.8)	51.8 ± 42.5	256 (55.8)	1.39 (1.24, 1.55)
University or above	376 (29.4)	51.0 ± 42.8	215 (57.2)	1.51 (1.33, 1.69)
Dental health education in school				
Yes	1024 (67.5)	52.2 ± 43.4	598 (58.4)	1.51 (1.41, 1.62) *
No	493 (32.5)	55.1 ± 47.3	265 (53.8)	1.32 (1.17, 1.47)
Teeth brush frequency				
≤ 1 time/day	587 (38.7)	54.5 ± 44.8	327 (55.7)	1.37 (1.24, 1.50)
≥ 2 times/day	930 (61.3)	52.3 ± 44.7	536 (57.6)	1.50 (1.39, 1.62)
Brush teeth after sugars intake				
Yes	166 (10.9)	45.8 ± 44.6 **	99 (59.6)	1.59 (1.30, 1.88)
No	1351 (89.1)	54.0 ± 44.7	764 (56.6)	1.43 (1.34, 1.53)
Use of dental floss or mouthwash				
Yes (one or both)	477 (31.4)	57.0 ± 47.4 *	277 (58.1)	1.48 (1.33, 1.64)
No (neither)	1040 (68.6)	51.4 ± 43.4	586 (56.3)	1.44 (1.33, 1.54)
Regular dental visiting				
Yes	318 (21.0)	53.2 ± 47.7	202 (63.5) **	1.67 (1.47, 1.88) **
No	1199 (79.0)	53.1 ± 44.0	661 (55.1)	1.39 (1.30, 1.49)

* *p* < 0.05, ** *p* < 0.01, *** *p* < 0.001; compared by Mann–Whitney U test, Kruskal–Wallis test or Chi-square test. DMFT: decayed, missing, and filled permanent teeth. BMI status: grouped by age- and sex- specific BMI cut-off values. Family monthly income: low (<5000 *RMB*), middle (5000–9000 *RMB*), high (>9000 *RMB*). *RMB*: Renminbi, Chinese official coupons, 1 *RMB* ≈0.16 *USD*.

**Table 2:** The average free sugars intake from food groups by DMFT score (number of decayed, missing, and filled permanent teeth) (mean ± *S.D.*, g/d).

The average intake	Total (*n* = 1517)	DMFT = 0 (*n* = 654)	DMFT ≥ 1 (*n* = 863)
**Food Groups**			
SSBs **	33.9 ± 40.6	30.6 ± 38.9	36.5 ± 41.6
Confections **	10.1 ± 22.8	8.3 ± 19.2	11.4 ± 25.2
Flavored milk/yogurt **	9.1 ± 15.2	8.1 ± 14.6	9.9 ± 15.6
Preserved fruits **	6.3 ± 18.3	5.6 ± 18.4	6.8 ± 18.2
Cakes or desserts	3.8 ± 7.9	3.3 ± 6.6	4.1 ± 8.8
Honey	2.5 ± 9.7	1.8 ± 5.8	2.9 ± 11.8
Total **	53.1 ± 44.7	48.2 ± 41.1	56.9 ± 47.0

* *p* < 0.05, ** *p* < 0.01, *** *p* < 0.001; comparison between DMFT = 0 group and DMFT ≥ 1 group by Mann–Whitney U test. SSBs include sodas, vegetable protein beverages, juice or juice drinks, tea drinks, sports drinks, and bubble tea.

**Table 3:** Binary logistic regression models of association between free sugar intake and dental caries experience among adolescents (DMFT = 0 as the reference).

Variables	Crude OR (95% CI)	Adjusted OR (95% CI)
Free sugars intake (vs. Low)		
Middle (25~50 g/d)	1.294 (0.988, 1.695)	1.248 (0.949, 1.641)
High (>50 g/d)	1.446 (1.138, 1.839) **	1.463 (1.146, 1.867) **
Sources of Free Sugars		
SSBs	1.004 (1.001, 1.006) **	1.005 (1.002, 1.007) **
Confections	1.007 (1.002, 1.012) *	1.005 (1.000, 1.010) *
Flavored milk/yogurt	1.008 (1.001, 1.015) *	1.007 (1.000, 1.014) *
Preserved fruits	1.004 (0.998, 1.010)	1.003 (0.997, 1.009)
Cakes or Desserts	1.014 (1.000, 1.028)	1.013 (0.999, 1.027)
Honey	1.014 (1.001, 1.028) *	1.015 (1.001, 1.029) *

* *p* < 0.05, ** *p* < 0.01, *** *p* < 0.001. OR, odds ratio. 95% CI, 95% confidence interval. Adjusted by sex, age, BMI status, domicile place, and regular dental visiting. SSBs include sodas, vegetable protein beverages, juice or juice drinks, tea drinks, sports drinks, and bubble tea.

## Data Availability

The data that support the findings of this study are not publicly available due to the data containing information that could compromise participant privacy but are available from the corresponding author on reasonable request.

## Appendix A

**Table A1:** Food item, quantity, and free sugars from Chinese Center for Disease Control and Prevention (China CDC).

Food Descriptor	Quantity	Types	Free Sugars (g)
Carbonated drinks	100 mL	9	9.79
Bubble tea	100 mL	7	7.89
Tea drinks	100 mL	6	4.31
Juice or juice drinks	100 mL	21	10.47
Vegetable protein beverages	100 mL	19	7.14
Sports drinks	100 mL	6	8.22
Flavored milk/yogurt	100 mL	29	12.28
Biscuits, cakes	100 g	118	17.50
Chocolate, confectionery	100 g	14	44.63
Fruit preserves	100 g	12	55.04
Honey	100 g	2	73.00

## Appendix B

**Table A2:** Age- and sex-specific BMI cut-off values for Chinese children and adolescents (6–18 years old, kg/m^2^).

Age (Years)	Boys		Girls	
	Overweight	Obese	Overweight	Obese
6.0	16.4	17.7	16.2	17.5
6.5	16.7	18.1	16.5	18.0
7.0	17.0	18.7	16.8	18.5
7.5	17.4	19.2	17.2	19.0
8.0	17.8	19.7	17.6	19.4
8.5	18.1	20.3	18.1	19.9
9.0	18.5	20.8	18.5	20.4
9.5	18.9	21.4	19.0	21.0
10.0	19.2	21.9	19.5	21.5
10.5	19.6	22.5	20.0	22.1
11.0	19.9	23.0	20.5	22.7
11.5	20.3	23.6	21.1	23.3
12.0	20.7	24.1	21.5	23.9
12.5	21.0	24.7	21.9	24.5
13.0	21.4	25.2	22.2	25.0
13.5	21.9	25.7	22.6	25.6
14.0	22.3	26.1	22.8	25.9
14.5	22.6	26.4	23.0	26.3
15.0	22.9	26.6	23.2	26.6
15.5	23.1	26.9	23.4	26.9
16.0	23.3	27.1	23.6	27.1
16.5	23.5	27.4	23.7	27.4
17.0	23.7	27.6	23.8	27.6
17.5	23.8	27.8	23.9	27.8
18.0	24.0	28.0	24.0	28.0

BMI, body mass index. BMI = weight/height^2^ (kg/m^2^).

## Appendix C

**Table A3:** Weekly consumption frequencies of free sugars in adolescents by DMFT groups (mean ± *S.D.*, times/week).

Food Groups	Total (*n* = 1517)	DMFT = 0 (*n* = 654)	DMFT ≥ 1 (*n* = 863)
SSBs **	6.2 ± 8.0	5.5 ± 7.1	6.7 ± 8.6
Confections **	1.7 ± 3.4	1.5 ± 3.1	1.9 ± 3.6
Flavored milk/yogurt **	2.4 ± 3.7	2.1 ± 3.3	2.6 ± 4.0
Preserved fruits **	0.8 ± 2.5	0.7 ± 2.0	0.9 ± 2.7
Cakes or desserts *	1.5 ± 2.8	1.4 ± 2.5	1.6 ± 3.0
Honey	0.5 ± 1.9	0.4 ± 1.3	0.6 ± 2.3
Total ***	13.1 ± 14.5	11.5 ± 11.9	14.4 ± 16.0

* *p* < 0.05, ** *p* < 0.01, *** *p* < 0.001; compared by Mann–Whitney U test. SSBs include sodas, vegetable protein beverages, juice or juice drink, tea drink, sports drink, bubble tea.
